# A Potential Way to Decrease the Know-Do Gap in Hospital Infection Control in Vietnam: *“Providing Specific Figures on Healthcare-Associated Infections to the Hospital Staff Can ‘Wake Them Up’ to Change Their Behaviour”*

**DOI:** 10.3390/ijerph15071549

**Published:** 2018-07-22

**Authors:** La Thi Quynh Lien, Eva Johansson, Pham Thi Lan, Nguyen Thi Kim Chuc, Nguyen Thi Minh Thoa, Nguyen Quynh Hoa, Ho Dang Phuc, Ashok J. Tamhankar, Cecilia Stålsby Lundborg

**Affiliations:** 1 Health Systems and Policy (HSP): Improving the Use of Medicines, Department of Public Health Sciences, Karolinska Institutet, Tomtebodavägen 18 A, 17177 Stockholm, Sweden; eva.Johansson@ki.se (E.J.); ejetee@gmail.com (A.J.T.); Cecilia.Stalsby.Lundborg@ki.se (C.S.L.); 2 Department of Pharmaceutical Management and Pharmaco-Economics, Hanoi University of Pharmacy, 13-15 Le Thanh Tong, Hoan Kiem District, Hanoi 110403, Vietnam; 3 Department of Family Medicine, Hanoi Medical University, 01 Ton That Tung, Dong Da District, Hanoi 116516, Vietnam; landhy2003@yahoo.com (P.T.L.); ntkchuc@yahoo.com (N.T.K.C.); ntmthoa@gmail.com (N.T.M.T.); 4 National Centralised Drug Procurement Centre, Vietnam Ministry of Health, 138A Giang Vo Street, Ba Dinh district, Hanoi 118401, Vietnam; quynhhoa29@gmail.com; 5 Institute of Mathematics-VAST, 18 Hoang Quoc Viet, Cau Giay District, Hanoi 122121, Vietnam; hodang54@yahoo.com; 6 Indian Initiative for Management of Antibiotic Resistance, Department of Environmental Medicine, R.D. Gardi Medical College, Agar Road, Ujjain 456006, India

**Keywords:** qualitative study, perceptions, hospital staff, infection control, healthcare-associated infection, know-do gap, Vietnam

## Abstract

Adequate infection control plays a key role in preventing healthcare-associated infections (HAIs). This study aimed to explore staff perceptions of hospital infection control in a rural and an urban hospital in Vietnam. Individual interviews were conducted with hospital managers, and focus group discussions were conducted with doctors, nurses and cleaning workers separately. Content analysis was applied. An interview guide including discussion points on HAIs, hand hygiene and healthcare waste management was used. Generally, the staff were knowledgeable of hospital infection control, but they were not aware of the situation in their own hospital, and infection control practices in the hospitals remained poor. Reported difficulties in infection control included lack of resources, poor awareness and patient overload. A main theme emerged: ‘Making data on HAIs available for health workers can improve their awareness and motivate them to put their existing knowledge into practice, thus decreasing the know-do gap in infection control’. This could be a feasible intervention to improve infection control practice in the hospitals with limited resources, high workload and patient overload.

## 1. Introduction

Poor infection control favours the spread of microorganisms in healthcare facilities and beyond, causing healthcare-associated infections (HAIs) [1,2]. HAIs are recognised as one of the most frequent adverse events for patients while receiving care [3,4]. HAIs result in prolonged hospital stays, a high financial burden on health systems, high costs for patients and their families, excess deaths and increased resistance of bacteria to antibiotics [1,5]. The risks of HAIs are significantly higher in low- and middle-income countries, and the effect on patients and healthcare systems is severe and greatly underestimated [6,7].

Vietnam, a lower middle-income country (LMIC), experiences high levels of HAIs, as well as antibiotic resistance [8,9]. Many Vietnamese hospitals are old and overcrowded, making effective infection control challenging. Occupancy can exceed 100%, particularly during communicable disease outbreaks [8]. The first regulation that mentioned hospital infection control was the Ministry of Health (MoH)’s regulation on hospital organisation, conditions and activities, issued in 1997 [10]. Since then, efforts have been made to improve infection control system, however, much is still unknown regarding compliance with the regulations.

In Vietnam, the healthcare system consists of four administrative levels: central hospitals, provincial hospitals and provincial healthcare centres, district hospitals and district healthcare centres, and commune healthcare centres. Central hospitals are under the direct control of the Ministry of Health, located in large cities providing medical services as top referral hospitals. Provincial/district hospitals and provincial/district healthcare centres are under the control of the provincial health departments and are responsible for the medical care of the people of the provinces and districts respectively. Commune healthcare centres are under the control of the district health departments and are responsible for the primary healthcare of the local people.

We conducted this qualitative study to explore staff perceptions of hospital infection control, including HAIs, hand hygiene, and healthcare waste management in a rural district hospital and an urban provincial hospital in Vietnam.

Qualitative research is usually used to gain an understanding of the problem or helps to develop ideas for potential quantitative research. It can also be used concurrently with quantitative research to confirm, compare or cross-validate the results [11]. In the case of our study, we use qualitative methods to provide insights on the issue of hospital infection control, as well as to compare and better understand findings from a quantitative study on the same topic conducted at the same hospitals [12].

## 2. Materials and Methods

A qualitative study, using individual interviews and focus group discussions (FGDs), was conducted with 50 hospital staff in total in a rural district hospital (220 beds) and an urban provincial hospital (520 beds) in Hanoi, Vietnam in 2013. The rural district hospital had 46 doctors, 110 nurses, 12 midwives and 12 cleaning workers at the time of the study, while the urban provincial hospital had 181 doctors, 392 nurses, 32 midwives, and 35 cleaning workers. For confidentiality reasons, no more details are given.

### 2.1. Data Collection and Participants

A semi-structured guide was developed, based on literature and previous experience of the authors, and consisted of discussion points to encourage greater detail, variety and clarity [13,14]. The discussion points were in relation to HAIs, hand hygiene and healthcare waste management (Table 1). The guide was pilot tested with a group of doctors in the rural hospital. Six interviews were conducted with leaders of the hospitals. Six FGDs were conducted with hospital staff according to occupation: one group of doctors, one group of nurses and one group of cleaning workers in each hospital (Table 2).

Purposive sampling was used to select the participants, ensuring that a range of views was captured from different hospital staff. Leaders of the hospitals were selected for individual interviews as they have an overview of the hospital activities. Hospital staff from different wards of the hospitals, who were directly involved in hospital practices related to infection control, were invited to participate in the study. No potential participants refused the participation. Participation was voluntary. The staff were informed about the aim of the studies and written consents were obtained. Interviews and discussions were carried out by one of two researchers (Pham Thi Lan/ Ho Dang Phuc) in locations chosen by the participants. A research assistant recorded and took notes during the discussions. None of the researchers or research assistants were employed at any of the two hospitals. The researchers and research assistants had no direct relationship to the participants. The interviews/discussions were continued until the researchers saw that no new information was brought up. The interviews lasted for 45–60 min each. The FGDs lasted between 60 and 120 min.

### 2.2. Data Analysis

Audio recordings were transcribed verbatim by two of the authors (La Thi Quynh Lien/ Nguyen Thi Minh Thoa) and checked by the first author (La Thi Quynh Lien). Data from transcripts were analysed manually using manifest and latent content analysis [15]. The text was read thoroughly several times to obtain a sense of the whole and then divided into “meaning units”, which are parts of the original transcript that carries a specific meaning relating to the objective of the study. Each meaning unit was condensed and labelled with a code. Coding and quotation selection were carried out in Vietnamese, then coding results and selected quotations were translated into English by the first author (La Thi Quynh Lien). This process was checked by the third author (Pham Thi Lan), fluent in both Vietnamese and English. Similar codes were grouped into categories. Examples of coding process are presented in Table 3.

Categories were discussed among all the authors until a main theme and sub-themes were identified. The authors have varied professional and research backgrounds (pharmacy, medicine, nursing, public health and environmental health).

### 2.3. Ethical Approval

This study was approved by Hanoi Medical University Review Board in Bio-Medical Research (No. 116/HMU IRB). The participants were informed that participation was voluntary, that they could withdraw from the study at any time, and that confidentiality would be maintained throughout.

## 3. Results

In the analysis, one main theme and three sub-themes emerged. The main theme identified was ‘Making data on HAIs available for health workers can improve their awareness and motivate them to put their existing knowledge into practice, thus decreasing the know-do gap in infection control’. The main theme, sub-themes and categories are presented in Table 4. Main findings are presented according to categories under each sub-theme. Participant quotes from different FGDs and interviews are used to illustrate the findings under each category. Within the quotes, explanations by the authors are given in square brackets.

### 3.1. Sub-Theme 1: Hospital Staff Were Knowledgeable of HAIs, but They Were not Aware of the HAI Situation in Their Hospitals

#### 3.1.1. Healthcare-Associated Infections

In general, the staff were knowledgeable of the HAI definition and types of HAIs. They pointed out different examples of HAIs including surgical site infections, hospital-acquired endometrial infections, suture infections after giving birth, hospital-acquired digestive disorders, catheter placement infections, hospital-acquired respiratory tract infections, hospital-acquired tuberculosis and hospital-acquired HIV through needle stick injuries.

“*An HAI, firstly, is an infection that the patients have acquired in the hospital or in another healthcare facility, secondly symptoms of infection, that were absent at the time of admission, have appeared 48 h after admission. *”

(FGD 5—nurses, rural hospital)

The participants were aware of the transmission ways of HAIs through hospital staff, patients, medical equipment with poor disinfection and sterilisation, hospital waste and the air in hospitals. Among the FGDs, the group of cleaning workers in the rural hospital were not aware of HAIs and their own role in preventing HAIs in the hospital. They supposed that the possibility of people getting infections in the hospital had nothing to do with them, and that it was the hospital’s responsibility. They expressed that they merely cared about the risks to themselves of getting infections due to their work.

“*When doctors use one pair of gloves for examination of different patients, infections can be transferred from one infected patient to others.*”

(FGD 3—cleaning workers, urban hospital)

“*We are mainly afraid that we get infections, so we wear gloves and protection masks, pay attention to injection needles…Our work is just cleaning and throwing away the waste. Regarding HAIs, that is the hospital’s responsibility.*”

(FGD 6—cleaning workers, rural hospital)

#### 3.1.2. Situation of Healthcare-Associated Infections

The hospital staff were not aware of the situation of HAIs in their hospitals. In the rural hospital, the staff perceived the HAI situation in different ways. While some participants supposed that in their hospital there were no, or very few, cases of HAIs, other staff argued that there could be some or even many cases. According to the participants, the hospital did not have adequate laboratory capacity for microbiological analysis, and the hospital staff did not know about any specific data on HAI. However, some participants acknowledged that cross sectional surveys on HAIs were conducted once per year in the hospital in order to improve infection control according to a regulation of the local Health Department.

One said: “*In fact, in our hospital there are almost no HAIs. The evidence is that the rate of surgical site infections is very low.*”

(FGD 4—doctors, rural hospital)

Another one argued: “*I don’t think that we have few cases of HAIs. We couldn’t detect them [HAIs] and we do not have statistical data.*”

(FGD 4—doctors, rural hospital)

In the urban hospital, the staff supposed that the HAI situation in their hospital was serious. They were of the opinion that the risks of getting HAIs were extremely high if the patients had been staying in the hospital for a long time.

“*Almost every patient staying for a long time, more than five days, gets HAI, for example patients with cerebrovascular accident, respiratory infection, catheter placement and patients who receive mechanical ventilation for a long time.*”

(FGD 2—nurses, urban hospital)

However, the participants emphasised that they were not informed about any specific HAI-related data, although cross-sectional surveys on HAIs were conducted every year in the hospital.

“*We cannot get data from anywhere … In the hospital there might be many HAIs in reality.*”

(FGD 1—doctors, urban hospital)

#### 3.1.3. Difficulties in Controlling HAIs

The participants acknowledged difficulties in infection control in their hospitals, including poor budget for infection control; lack of facilities and insufficient equipment and supplies; lack of staff specialised in infection control; patient overload; poor awareness of hospital staff, as well as patients and their relatives. In the hospitals, no staff received education specified in infection control, and in fact no one wanted to work with infection control. The rural hospital merely had a group in charge of infection control, which mainly took care of laundry, drying and steaming clothes, linen and waste management. The hospital did not have a microbiological laboratory.

“*We have to use one oxygen generator with the same oxygen tube during 2–3 days, even 10 days for several patients, causing high risks for getting HAIs.*”

(FGD 3—doctors, rural hospital)

“*We cannot culture and test susceptibility of bacteria due to the absence of a microbiological laboratory, so we cannot make conclusions about suspected cases if they are HAIs or not.*”

(FGD 4—nurses, rural hospital)

Poor awareness of hospital staff emerged as a main challenge in infection control. Certain efforts in improving infection control in the hospitals already existed, for example, providing alcohol-based hand rub solution, annual training on infection control, and availability of standard protocols; however, the practices remained poor. The participants supposed that the staff might think about infection control as someone else’s issue but not his or hers. There is a need to make all hospital staff, starting from the heads of wards, understand the HAI situation in their hospital and view infections control as their concern.

“*We do training every year but the practices (regarding infection control) are still* poor.”

(II 2—a hospital manager, urban hospital)

“*Standard procedures exist but are not followed. Trainings are organised but the practices remain poor. Providing specific figures on HAIs to the hospital staff can ‘wake them up’ to change their behaviour.*”

(II 5—a hospital manager, rural hospital)

Patient overload and poor awareness of patients and their relatives were common challenges in infection control in both the hospitals. It was emphasised in the rural hospital that even in the department of Infectious Diseases, patients had to share their beds, which was a high risk for infection transmission. The participants also acknowledged that patients and their relatives did not comply with hygiene guidelines and procedures, making infection control more difficult.

### 3.2. Sub-Theme 2: Hospital Staff Were Aware of the Importance of Hand Hygiene in Preventing Healthcare-Associated Infections, But They Acknowledged Poor Hand Hygiene Practices in Their Hospitals

#### 3.2.1. Importance of Hand Hygiene

The hospital staff were aware of the importance of hand hygiene in preventing HAIs. The participants acknowledged that all health measures in hospitals were hand-related; therefore, if hand hygiene procedures were complied with, the incidence of HAIs could be reduced significantly.

“*Hand hygiene is very important because it contributes to preventing HAIs. If everybody followed hand hygiene procedures, the rate of HAIs could be reduced a lot.*”

(FGD 5—nurses, rural hospital)

#### 3.2.2. Reasons for Noncompliance

When discussing the reasons for noncompliance with hand hygiene, patient overload was strongly emphasised by the participants in both the hospitals. The doctors viewed the large number of patients they had to examine every day as a dominant obstacle for them in following the hand washing procedures.

“*Examining 100–150 patients per day, we do not even find time to put our heads up to see the faces of the patients, not to mention carrying out hand-washing procedures.*”

(II 1—a hospital manager, urban hospital)

“*Here in our hospital a doctor may have to examine about 70, even 100 patients in the morning. If you count 30 s per patient to wash the hands, for 100 patients—50 min excluding time for moving and wiping hands. It is impossible to follow.*”

(FGD 4—doctors, rural hospital)

The participants viewed the lack of facilities as another difficulty in compliance with hand hygiene. They stated that the hospitals did not provide sufficient numbers of sinks for hand washing, soap and antiseptic liquid. However, it emerged that sometimes even when the necessary facilities and materials were available for hand hygiene, the practices were still poor.

“*Sometimes liquid for hand washing is available, but they do not use.*”

(FGD 1—doctors, urban hospital)

### 3.3. Sub-Theme 3: Hospital Staff Acknowledged the Importance of Healthcare Waste Management, but They Were Not Aware of Healthcare Waste Treatment in Their Hospitals

#### 3.3.1. Healthcare Waste Management

The staff were aware of the importance of healthcare waste management not only in preventing HAIs, but also in inhibiting or minimising the spread of diseases to the community. The participants provided various examples for the ways of getting HAIs from hospital waste, including that infections can be transmitted from hospital waste through flies and mosquitoes, and that HIV and hepatitis B can be transmitted through needle stick injuries.

“*With bandages, injection needles used for HIV or hepatitis B patients, if we do not classify well, we may touch them by chance, and it is easy to get infections … Two years ago in the Department of Emergency there was such a case. When a nurse was giving an anesthetic injection to a HIV positive patient, unfortunately she got punctured on her hand.*”

(FGD 1—doctors, urban hospital)

The hospital staff were generally well informed about healthcare waste classification. During the FGDs, groups of nurses and cleaning workers discussed this in more detail than the groups of doctors. The staff were aware of different types of bags and containers for respective types of waste.

“*Healthcare waste—in yellow bags, domestic waste—green bags, hard things in black bags and white bags for recycled waste … Sharp things are put into yellow boxes, and then the boxes are put into black bags.*”

(FGD 6—cleaning workers, rural hospital)

However, it emerged that many hospital staff members were not aware of the healthcare waste treatment in their hospitals. They seemed to not pay attention and not care about it, and therefore did not know how healthcare waste was treated in the hospitals.

“*In our department, we only do waste classification, put it in the right bags, then we do not know where they are taken and treated.*”

(FGD 2—doctors, rural hospital)

#### 3.3.2. Situation of Healthcare Waste Management

From the participant views, waste management seemed to be better in the urban hospital than in the rural hospital. In the urban hospital, the participants acknowledged that waste management was implemented well, especially healthcare waste classification. According to them, this was because the hospital, some years ago, was put on the list of the hospitals causing serious environmental pollution. As a consequence, since then, the hospital has taken healthcare waste management seriously. In the rural hospital, various participants acknowledged the role of patients and their relatives in the hospital waste management. They reported that the noncompliance of patients and their relatives with waste management procedures made waste management in the hospital more challenging.

“*Patient relatives throw waste everywhere. They even throw big diapers used by patients into the drains, clogging the drains.*”

(FGD 3—doctors, rural hospital)

## 4. Discussion

To our knowledge, this is the first study from Vietnam that explores perceptions among various hospital staff with regard to infection control, including HAIs, hand hygiene and healthcare waste management. 

### 4.1. Lack of Resources for Infection Control

It has been reported previously that lack of resources for infection control is one of the major challenges for resource-limited countries [16,17,18,19]. In Vietnam, as in many other LMICs, the overall investment allocated for healthcare is limited. While high-income countries spent up to 17% of the Gross Domestic Product (GDP) on healthcare in 2013, the Government of Vietnam spent only 6.0% of GDP on healthcare (there has been a decrease from 6.4 to 6.0 since 2010) [20]. These scarce healthcare resources may explain the difficulties in infection control in the studied hospitals including lack of facilities, insufficient equipment and supplies.

Our findings indicate that the rural district hospital had poorer conditions for infection control than the urban provincial hospital, which was highlighted by the lack of a microbiological laboratory, and a separate infection control department. In Vietnam, many public hospitals are still dependent mainly on the state budget [21]. The state budget is transferred to the hospitals in the form of line-item allocations from government health authorities, the rate of provision depending on the wealth of each province. The hospitals at the central and provincial level, or those located in wealthier regions, normally receive more investment and are better equipped with technology. A survey of infection control conducted in 51 hospitals including central, provincial and district hospitals in Vietnam indicated that there were still large differences between the central and local hospitals, especially district hospitals, not only in regard to infection control, but also in many other medical aspects. This is one of the existing problems of medical care in Vietnam [22].

### 4.2. Cleaning Workers’ Poor Understanding of Controlling HAIs

In this study, cleaning workers were not well aware of their role in preventing HAIs. A cross-sectional study, conducted in the same hospitals, indicated lower infection control knowledge as well as practice scores of cleaning workers compared to doctors and nurses [12]. In fact, there is evidence supporting that the role of cleaning in hospitals is an important intervention in controlling HAIs [23]. In the hospitals investigated, as in many other hospitals in Hanoi, cleaning workers were hired from a cleaning company and they received training merely by the cleaning company before they started working in the hospital. Therefore, there is a need for the hospital to improve cleaning workers’ understanding of their crucial role in controlling HAIs.

### 4.3. Patients and Their Relatives’ Poor Adherence to Hygiene Guideline and Procedures

Poor adherence of patients and their relatives to hygiene guides and procedures of the hospitals was reported as a difficulty in infection control. This seemed to be due to their lack of awareness of HAIs and infection control strategies. Interventions, for example, posters and reminders in patient rooms, can be applied in order to improve awareness and behaviour [14]. Moreover, a good awareness of infection control strategies, for example hand hygiene, can motivate patients to engage in improving hospital staff’s compliance to reduce the burden of HAIs. It has been shown in a study that patients who had information on HAIs were more likely to report that they were comfortable in asking a nurse or doctor to wash their hands or to wear gloves and masks before examining them [24].

### 4.4. The Know-Do Gap

The know-do gap in infection control practice has previously been reported in various studies [25,26,27,28]. A systematic review indicated that despite adequate knowledge of hand hygiene and the introduction of many initiatives, adherence to hand hygiene remains poor. Hand hygiene was considered to be an acquired habit rather than a reasoned process. This area is currently understudied and effective methods for changing health workers behaviours are needed to reduce HAIs and improve patients’ safety [29].

In this study, hospital staff were aware of the importance of hand hygiene in preventing HAIs. However, even when the necessary facilities were in place, they did not seem to comply with recommended hand hygiene procedures. A study conducted in nine hospitals in Vietnam indicated that the overall compliance with hand hygiene procedures was only 13.4% [30]. Suboptimal compliance to infection control precautions has been also reported in neighbouring countries of Vietnam like Thailand and Cambodia [31,32].

In our study, doctors complained that patient overload was a dominant barrier for their compliance. However, if alcohol-based hand rub was available, it would be feasible for them to use it after each patient. Contradictions between what doctors knew and what they actually did were also reported from a high-income country with a well-developed healthcare system [33]. According to that study, doctors were knowledgeable about correct hand hygiene practices, but they failed to recognize that their own practices might be harmful to patients. It emerged from our findings that one determinant of this know-do gap might be the staff’s poor awareness of the HAI problem in their own hospitals.

### 4.5. Measures to Improve Infection Control Practice

Measures to improve infection control practice including training, proper supply of facilities, frequent audit and control were suggested in previous studies [34,35]. However, these are not easy to implement in the studied hospitals as well as in many other Vietnamese hospitals which are old, resource-limited, overcrowded and with high workload. Of note is that, in the studied hospitals, surveillance on HAIs was carried out every year, but the staff were not aware of any data on HAIs in their hospitals. If hospital staff were aware of the situation of HAIs in their hospital, they might become ‘awake’, meaning that they would become aware of the need for behaviour change, and the know-do gap could be diminished.

### 4.6. Methodological Consideration

The study was conducted in different types of hospitals in terms of their catchment area (urban and rural) and their level in the healthcare system (district and provincial), which allows insights into understanding the existing healthcare system. Both individual interviews and FGDs were used for data collection. Individual interviews with the leaders of the hospitals provided a perspective from the management point of view, while FGDs with hospital staff, who were directly involved in the day to day hospital activities, helped for open-minded and flexible discussions and provided a detailed picture. FGDs were conducted with various groups of health workers including doctors, nurses and cleaning workers, which would bring out diverse insights.

To improve the credibility of the findings, triangulation and cross-checking were applied during data analysis, as well as during result interpretation [36]. Both manifest and latent analysis were used. The researchers have different backgrounds with extensive experience in their own fields, bringing various perspectives, which is important when analysing qualitative data. Cross-checking of the interview and discussion transcripts by two researchers could minimize misinterpretation. None of the researchers were employed at the studied hospitals, and the researchers and research assistants conducting FGDs and interviews had no direct relationship to the participants, ensuring reflexivity of the study [36].

The study has some limitations. Not all authors understand Vietnamese. During data analysis, the coding and quotation selection were performed in Vietnamese and then coding results and selected quotations were translated into English, which made the analysis process more time consuming. Another limitation is that the member check of the transcripts was not done by the participants, as it was difficult to gather the discussion groups.

Although the results of this study cannot be generalised in a numerical manner, we believe that the study and its results can be of value in many other similar settings.

## 5. Conclusions

The staff were generally knowledgeable of hospital infection control, but they were not aware of the situation in their own hospital, and infection control practices in the hospitals remained poor. Reported difficulties in infection control were lack of resources, poor awareness and patient overload. Making data on HAIs available for health workers can be a feasible intervention to improve infection control practices in the hospitals with limited resources, where optimising facilities and systematic approaches in improving infection control are difficult.

## Figures and Tables

**Table 1:** Interview/Discussion guide.

Interview/Discussion Guide
- The definition of healthcare-associated infections (HAIs) - Types of HAIs - Transmission ways of HAIs - Situation of HAIs in the hospitals - Difficulties in controlling HAIs - Importance of hand hygiene - Reasons for noncompliance with hand hygiene guidelines - Healthcare waste management Situation of healthcare waste management in the hospitals

HAIs: Healthcare-associated infections.

**Table 2:** Characteristics of participants in individual interviews and focus group discussions in a rural and an urban hospital in Vietnam.

Interview Code	Hospital	Category of Staff	Number of Participants		Number of Years of Experience
			Male	Female	
II 1	Urban	Hospital manager	-	1	28
II 2	Urban	Hospital manager	-	1	27
II 3	Urban	Hospital manager	1	-	31
II 4	Rural	Hospital manager	1	-	26
II 5	Rural	Hospital manager	1	-	14
II 6	Rural	Hospital manager	1	-	32
FGD 1	Urban	Doctors (Dept. of Neurology, Dept. of General Internal Medicine, Dept. of Emergency and Intensive Care, Dept. of Occupational Diseases)	3	3	1–25
FGD 2	Urban	Nurses (Dept. of Surgery, Dept. of General Internal Medicine, Dept. of Emergency and Intensive Care, Dept. of Nursing)	2	5	1–27
FGD 3	Urban	Cleaning workers (Dept. of Emergency and Intensive Care, Dept. of Cardiology, Dept. of Surgery, Dept. of Endocrinology, Dept. of General Internal Medicine, Dept. of Gastroenterology, Public area, Cleaning Administrator)	-	9	1–12
FGD 4	Rural	Doctors (Dept. of Surgery, Dept. of Obstetrics, Dept. of Pediatrics, Dept. of Ophthalmology Otorhinolaryngology, Dept. of Emergency and Intensive Care, Dept. of General Planning)	5	3	1–20
FGD 5	Rural	Nurses (Dept. of Nursing, Dept. of Infectious Diseases, Dept. of Surgery, Dept. of Ophthalmology and Otorhinolaryngology, Dept. of General Examination, Dept. of Emergency and Intensive Care, Dept. of Traditional Medicine, Dept. of Obstetrics)	1	7	1–32
FGD 6	Rural	Cleaning workers (Administration area, Dept. of Imaging and Radiology, Dept. of Surgery, Public area, Dept. of Traditional Medicine, Dept. of Emergency and Intensive Care)	-	6	1–2
Total			15	35	

II: Individual interview; FGD: Focus group discussion; Dept.: Department.

**Table 3:** Examples of coding process.

Condensed Meaning Units	Codes	Category
- HAIs are the infections occurring in patents in the hospital after admission - During stay in the hospital, patients can get infections	Occurring in the hospital	Healthcare-associated infections
- There are infections incubating for 5 days, 7 days and even longer, so the infections may appear after discharge	Appearing after discharge	
- Surgical site infections; hospital-acquired endometrial infections; stitches infection after giving birth - Nosocomial digestive disorders; catheter placement infection; nosocomial tuberculosis	Examples of HAIs	
- Noncompliance with hand hygiene - Doctors don’t wash their hands after each patient - Not absolute compliance with aseptic discipline	Through hospital staff	
- Through needle puncture or bandage - Sharing bows or cups	Through medical equipment and domestic utensils	
- HIV and hepatitis B can be transmitted through needle puncture - Hospital waste can be a source of diseases - Hospital waste if not properly managed can lead to HAIs	Through hospital waste	
- Infected air; tuberculosis cases from one patient to another - Respiratory infections can be transmitted within the hospital	Through the air	

HAIs: Healthcare-associated infections; ICU: Intensive Care Unit.

**Table 4:** The main theme, sub-themes and categories.

**Main Theme**	Making data on HAIs available for health workers can improve their awareness and motivate them to put their existing knowledge into practice, thus decreasing the know-do gap in infection control		
**Sub-themes**	**Sub-theme 1**	**Sub-theme 2**	**Sub-theme 3**
	Hospital staff were knowledgeable of HAIs, but they were not aware of the HAI situation in their hospitals	Hospital staff were aware of the importance of hand hygiene in preventing HAIs, but they acknowledged poor hand hygiene practices in their hospitals	Hospital staff acknowledged the importance of healthcare waste management, but they were not aware of healthcare waste treatment in their hospitals
**Categories**	- HAIs - Situation of HAIs - Difficulties in controlling HAIs	- Importance of hand hygiene - Reasons for noncompliance	- Healthcare waste management - Situation of healthcare waste management

HAIs: Healthcare-associated infections.
